# Cannabidiol Exposure During Rat Pregnancy Leads to Labyrinth-Specific Vascular Defects in the Placenta and Reduced Fetal Growth

**DOI:** 10.1089/can.2023.0166

**Published:** 2024-06-18

**Authors:** Sofia Allen, Bryony V. Natale, Alexis O. Ejeckam, Kendrick Lee, Daniel B. Hardy, David R.C. Natale

**Affiliations:** ^1^ Department of Biomedical and Molecular Sciences, Queen's University, Kingston, Ontario, Canada.; ^2^ Department of Obstetrics and Gynaecology, Queen's University, Kingston, Ontario, Canada.; ^3^ Department of Physiology and Pharmacology, The University of Western Ontario, London, Ontario, Canada.; ^4^ The Children's Health Research Institute, The University of Western Ontario, London, Ontario, Canada.; ^5^ Lawson Health Research Institute, The University of Western Ontario, London, Ontario, Canada.; ^6^ Department of Obstetrics and Gynaecology, The University of Western Ontario, London, Ontario, Canada.

**Keywords:** cannabidiol, fetal growth restriction, labyrinth zone, glucose transporter 1, pregnancy

## Abstract

**Introduction:**

Cannabis use is increasing among pregnant people, and cannabidiol (CBD), a constituent of cannabis, is often perceived as “natural” and “safe” as it is non-intoxicating. *In utero*, cannabis exposure is associated with negative health outcomes, including fetal growth restriction (FGR). The placenta supplies oxygen and nutrients to the fetus, and alterations in placental development can lead to FGR. While there has been some investigation into the effects of Δ^9^-THC, there has been limited investigation into the impacts of *in utero* gestational CBD exposure on the placenta.

**Methods:**

This study used histological and transcriptomic analysis of embryonic day (E)19.5 rat placentas from vehicle and CBD (3 mg/kg intraperitoneal injection) exposed pregnancies (E6.5-18.5).

**Results:**

The study revealed that pups from CBD-exposed pregnancies were 10% smaller, with the placentae displaying a decreased fetal blood space perimeter-to-area ratio. The transcriptomic analysis supported compromised angiogenesis and blood vessel formation with downregulated biological processes, including tube morphogenesis, angiogenesis, blood vessel morphogenesis, blood vessel development and vasculature development. Further, the CBD-exposed placentas displayed changed expression of glucose transporters (decreased GLUT1 and GR expression and increased GLUT3 expression). Transcriptomic analysis further revealed upregulated biological processes associated with metabolism. Finally, histological and transcriptomic analysis revealed altered cell populations within the placenta, specifically to syncytiotrophoblast layer II and endothelial cells.

**Conclusion:**

Together these results suggest that the structural changes in CDB-exposed placentae, including the altered expression of nutrient transporters and the changes to the placental fetal vasculature, may underlie the reduced fetal growth.

## Introduction

Cannabis contains hundreds of cannabinoid and noncannabinoid compounds, with the two main constituents being Δ^9^-tetrahydrocannabinol (Δ^9^-THC, the major psychoactive component) and cannabidiol (CBD, the largest nonintoxicating constituent).^1^ After the North American legalization of cannabis, the frequency of its use has significantly increased, including among pregnant people.^2,3^ Clinical studies indicate that the prevalence of cannabis exposure during pregnancy varies from 2% to 10%,^4–6^ with a disproportionate increase in young, urban, socioeconomically disadvantaged subpopulations.^7–9^ Reasons for cannabis use during pregnancy include recreational purposes along with self-treatment of pregnancy-related side effects, including depression and anxiety.^10^ However, the literature surrounding the short- and long-term effects of prenatal cannabis use is limited, with human studies complicated by confounding factors, including socioeconomic status and the use of multiple drugs.^11^

Among the pregnant population, self-treatment with CBD for nausea, pain, anxiety, and depression is on the rise and in part, this is thought to be owing to its large safety profile and that it is nonintoxicating, leading to a perception that it is “safe.”^12^ However, another variable that may soon contribute to CBD use in pregnancy is that it is actively being investigated and/or promoted as a treatment/potential treatment for a range of conditions, including anxiety, Crohn's disease, depression, diabetes, epilepsy, pain, post-traumatic stress disorder, and sleep disorders.^12–14^ As many of these conditions also affect pregnant people, CBD may more frequently become an essential part of a patient's proactive treatment plan; thus, it is crucial to understand if *in utero* exposure is safe in pregnancy, with focus on placental development and fetal outcome.

Despite some conflicting results, growing evidence supports an association between gestational cannabis exposure and low-birth-weight outcomes,^14^ which is of concern, given low birth weight can indicate a suboptimal *in utero* environment.^15–17^ Furthermore, low-birth-weight outcomes are associated with the development of noncommunicable diseases later in life.^18^ Animal studies have complemented those in humans by eliminating or isolating factors confounding clinical studies and elucidating novel mechanisms that can be further investigated in humans. Although several animal studies have demonstrated that gestational Δ^9^-THC exposure leads to fetal growth restriction (FGR),^19,20^ there are limited studies investigating the impact of CBD. Despite Wanner et al. recently demonstrating that female rat offspring exposed to CBD during gestation exhibit increased anxiety, improved memory, and changes to the epigenome in the brain, the effects of CBD on the placenta and fetal growth remain under-explored.^21^

We have previously demonstrated *in utero* Δ^9^-THC exposure in the rat (3 mg/kg intraperitoneal [i.p.] resulted in FGR and placental insufficiency).^19^ We identified structural and vascular placental defects at E19.5, whereby Δ^9^-THC exposed placentae had increased maternal blood space with a corresponding reduction in fetal blood space area and reduced labyrinth-specific expression of the glucose transporter GLUT1.^19^ Furthermore, using BeWo cells as a model of human cytotrophoblast,^22,23^ we showed that *in vitro* treatment with Δ^9^-THC led to reduced expression of *GLUT1* mRNA.^19^ As Δ^9^-THC and CBD share some signaling pathways (reviewed by Rokeby et al.),^24^ we sought to investigate whether CBD had the same effect on fetal growth and placental development. In this study, we assessed the effect of *in utero* CBD exposure during a similar exposure window, dose, and route of delivery as our previous study.^19^

To evaluate whether CBD had a similar impact on fetal growth and placental development, pups and placentae were evaluated at E19.5 with focus on labyrinth development and the cell populations and components associated with fetal capillaries within the placenta. We found that, like Δ^9^-THC, gestational CBD exposure reduced fetal growth and altered the perimeter-to-area ratio in the fetal capillaries. Furthermore, CBD exposure altered the expression of placental glucose transporters. Collectively, these data suggest that caution should be exercised when using or prescribing CBD during pregnancy.

## Materials and Methods

### Animals and experimental paradigm

All procedures were performed according to guidelines set by the Canadian Council on Animal Care with approval from the Animal Care Committee at The University of Western Ontario. Pregnant female Wistar rats (250 g) were purchased from Charles River (La Salle, St. Constant QC, Canada), shipped at embryonic day (E) 3, and left to acclimatize to the environmental conditions of the animal care facility for 3 days. For the entire experimental procedure, dams were maintained under controlled lighting (12:12 L:D) and temperature (22°C) with *ad libitum* access to food and water.^25^ In rats, exposure to 3 mg/kg of CBD administered i.p. leads to serum concentrations of 9 ng/mL.^26^ The 3 mg/kg of CBD (i.p.) dose was used to reflect the low end of the range of CBD reported in human umbilical cord tissue (10–335 ng/mL) from fetuses exposed to cannabis during pregnancy.^27^ Dams were randomly assigned to receive a daily dose of vehicle (VEH; 1:18 cremophor:saline i.p.) or CBD (3 mg/kg i.p.; Cayman Chemicals) from E6.5 to E18.5 (*N*=12 total, *N*=6 dams/group for E19.5 analysis). For all pregnancy outcomes, the dam/litter was the statistical unit.

### Placenta collection and preparation

Pregnant dams were killed using an overdose of pentobarbital (100 mg/kg i.p.), followed by decapitation at E19.5. Uterine tissue was examined to determine resorption numbers and litter size, and the fetuses were weighed. Two placentae per dam were dissected, trimmed, weighed, and collected for histological assessment and an additional two for RNA sequencing. One to two placentae/litter were randomly selected and bisected with one-half processed for histological examination (fixed in 4% paraformaldehyde/phosphate-buffered saline [PBS] overnight, washed in PBS, dehydrated through an ethanol series and paraffin-embedded as previously described)^19,28–30^ and another half processed for RNA extraction, as described hereunder.

### Immunohistochemistry

All histological assessments were performed on 5 μm sections (Leica microtome) on randomly selected slides from each treatment group (*n*=7), with a minimum of one placenta selected from each litter. Immunohistochemistry (IHC) was performed per the manufacturer's published protocol (Immpress Horse Anti Rabbit IgG Kit; Vector Labs). In brief, antigen retrieval (Citra Buffer; Biogenex) was performed in a 2100-Retriever (Electron Microscopy Science). Primary antibodies were diluted in 1×PBS +0.1% bovine serum albumin, incubated overnight at 4°C, visualized using Dako DAB according to the manufacturer's protocol (Dako) and counterstained with hematoxylin (Gills #2; Sigma). All IHC was conducted with their respective negative controls (omission of primary antibody).

Placentae were imaged using an EVOS M7000 Imaging System (Life Technologies). 40×images were taken using the M7000 scan and stitch function. 400×labyrinth-specific images excluded the junctional zone and fetal membranes, with one image taken in the labyrinth's center and the remaining images taken midway between the center and outside the region. In cases where a maternal blood canal was present, images were taken to the left or the right of the canal to exclude it from the image. IHC antibodies included: pericyte, endothelial, and syncytiotrophoblast layer II (SynTII) populations, αSMA (1:300, ab124964; Abcam),^19,28^ CD31 (1:500, ab182981; Abcam),^31^ and MCT4 (1:500, ab3314P; Millipore)^32,33^; proliferation, Ki67 (1:100, ab16667; Abcam)^19,28^; extracellular matrix components,^19^ fibronectin (1:200, ab23750; Abcam) and laminin (1:200, ab11575; Abcam); glucose transport, GLUT1 (1:300, ab652; Abcam),^19^ its upstream regulator, glucocorticoid receptor (GR) (1:200, 24050-1AP; Protein-tech),^19^ and GLUT3 (1:100, ab41524; Abcam).^34^

### Histological quantification and analysis

Ki67 and GR IHC: six nonoverlapping labyrinth-specific 400×images/placenta were captured, and positive cells (stained nuclei) were counted with data presented as the number of positive cells/field of view (FOV).^19,28^ αSMA, CD31, MCT4, GLUT1, laminin, and fibronectin IHC (400×magnification) staining was quantified using Celleste Imaging software (Life Technologies) on six nonoverlapping labyrinth-specific images/placenta with stained area measured and presented as a percentage of the FOV).^19,28^ GLUT3 (40×magnification) staining was quantified with the stained area measured and presented as a percentage of the fetal-derived placenta. As MCT4 stains the SynTII cells that are specific to the labyrinth layer, these slides were also used for manual measurements of the areas of the labyrinth layer (defined as the layer with positive MCT4 staining) and the junctional zone (as defined by the parietal trophoblast giant cells [TGC]) and presented as a percentage of the total fetal-derived placenta.^19,28–30^

Blood space analysis was performed with CD31-positive staining to identify the fetal endothelial cells (fetal capillaries, herein referred to as fetal blood spaces). In contrast, the CD31-negative blood spaces, associated with a sinusoidal TGC [S-TGC] as identified by their large nuclei), were identified as maternal blood spaces. For both maternal and fetal blood spaces, the area and perimeter were collected with data presented as area and perimeter:area ratio.^19,28–31^ Statistical analysis was performed using an unpaired *t*-test (Prism 9 software), with significance set at *p*<0.05. The data presented are expressed as normalized mean values±standard error of the mean. A single observer, blinded to experimental conditions, performed all assessments/quantification.

### Bulk RNAseq

At the time of dissection, half of each placenta was stored in RNAlater and frozen (−80°C). Genome Quebec performed RNA extraction for library construction and subsequent bulk RNA sequencing. In brief, total RNA was isolated using Qiagen RNeasy Kit (Qiagen), quantified, and its integrity was assessed using 5K/RNA/Charge Variant Assay LabChip and RNA Assay Reagent Kit (PerkinElmer). Libraries were generated from 250 ng of total RNA as follows: mRNA enrichment was performed using Illumina Stranded mRNA Prep (Illumina); adapters and polymerase chain reaction primers were purchased from IDT; libraries were quantified using the KAPA Library Quantification Kits—Complete kit (Universal) (Kapa Biosystems); average size fragment was determined using a LabChip GX II (PerkinElmer) instrument. The libraries were normalized, pooled, denatured in 0.02 N NaOH, and neutralized using HT1 buffer. The pool was loaded at 175 pM on an Illumina NovaSeq S4 lane using Xp protocol per the manufacturer's recommendations.

The run was performed for 2×100 cycles (paired-end mode). A phiX library was used as a control and mixed with libraries at a 1% level. Base calling was performed with RTA v3. Program bcl2fastq2 v2.20 was used to demultiplex samples and generate fastq reads. Fastq data files were analyzed using Partek Flow (St. Louis, MO) in collaboration with Dr. David Carter (Robarts Research Institute, Western University). After importation, data were aligned to the *Rattus norvegicus* rn7 genome using STAR 2.7.3a and annotated using rn7. Features with more than 26 reads were normalized using DESeq2. DESeq2 was also used to create fold change and *p*-values between groups. A filtered gene list was generated with genes that met the criteria of ≥1.5-fold change and an false discovery rate (FDR)-adjusted *p*-value (*q*-value) of ≤0.05.

This list was imported into Metascape (https://metascape.org) to identify statistically enriched down- and upregulated gene ontology (GO) biological processes using the Custom Analysis tool. Based on the Metascape analysis system, accumulative hypergeometric *p*-values and enrichment factors were calculated and used for filtering, with the remaining significant terms hierarchically clustered based on kappa-statistical similarities among their gene memberships. A 0.3 kappa score was applied as the threshold to cast the tree into term clusters, and a network layout was generated using a Metascape-generated subset of representative terms from the entire cluster. The network was visualized with Cytoscape with a “force-directed” layout and is edge bundled for clarity. One term from each cluster is selected to have its term description shown as a label.

## Results

### CBD exposure during pregnancy results in reduced fetal weight

*In utero,* gestational CBD exposure did not significantly alter the litter size, the litter size after resorptions or the number of resorptions (Table 1). Nor did exposure alter maternal food intake or maternal weight gain (data not shown). However, at E19.5, the fetuses from the CBD-exposed pregnancies were ∼10% smaller than those from the vehicle control group (Table 1). The fetal placental ratio can be used as a measure of placental efficiency and can be associated with pregnancy complications and placental pathology, where the associations can differ from those of fetal and placental weights alone.^15^ However, neither the placental nor the fetal-to-placental weight ratio was significantly altered between the CBD and VEH groups (Table 1).

**Table 1.** Fetal and placental measures at E19.5

	**Vehicle±SEM**	**CBD±SEM**	***p***
Litter size, *n*	12.17 ± 0.601	11.17 ± 1.078	0.436508
Litter size after resorption, *n*	11.33 ± 0.843	9.833 ± 1.493	0.402110
Resorptions, *n*	0.8333 ± 0.307	1.333 ± 0.558	0.450574
Fetal weight, g	**1.647 ± 0.038**	**1.474 ± 0.036**	**0.0017**
Placental weight, g	0.478 ± 0.021	0.465 ± 0.020	0.6609
Fetal:placental weight ratio	3.527 ± 0.107	3.293 ± 0.162	0.2347

Fetal growth in pregnancies exposed to 3 mg/kg CBD during gestation is reduced, whereas litter metrics are not altered. *n*=6 litters per treatment group. Student's *t*-test with significance identified in bold when *p*<0.05.

CBD, cannabidiol; SEM, standard error of the mean.

Although there was no change in placental weight, owing to the smaller fetus size, we assessed whether there were structural changes to the placentae,^19,28^ by assessing labyrinth and junctional zone size and the ratio between them as a measure of disruption in the relative proportion of the labyrinth.^28,35–37^ The labyrinth and junctional zone areas and their ratio were not significantly altered in the placentae from the CBD-exposed pregnancies relative to the VEH placentae (Table 2). Proliferation in the labyrinth typically peaks at mid-gestation and drops to a basal level by E16.5.^38^ However, as proliferation was increased in the placentae from Δ^9^-THC exposed pregnancies,^19^ proliferation was assessed, as indicated by Ki67 staining, although it was not altered by CBD exposure (Table 2).

**Table 2.** Placental measures at E19.5

	**Vehicle±SEM**	**CBD±SEM**	***p***
Labyrinth area, %	81.964 ± 0.741	80.010 ± 0.856	0.1100
Junctional zone area, %	18.036 ± 0.741	19.990 ± 0.856	0.1100
Labyrinth junctional zone ratio	4.599 ± 0.223	4.061 ± 0.230	0.1188
Ki67 (#/FOV)	102.80 ± 9.488	97.600 ± 10.211	0.7156

Placental layers and proliferation in placentae from pregnancies exposed to 3 mg/kg CBD during gestation is not altered.

FOV, field of view.

### Fetal capillary perimeter:area ratio is reduced in placentae from CBD-exposed pregnancies

**FIG. 1.**
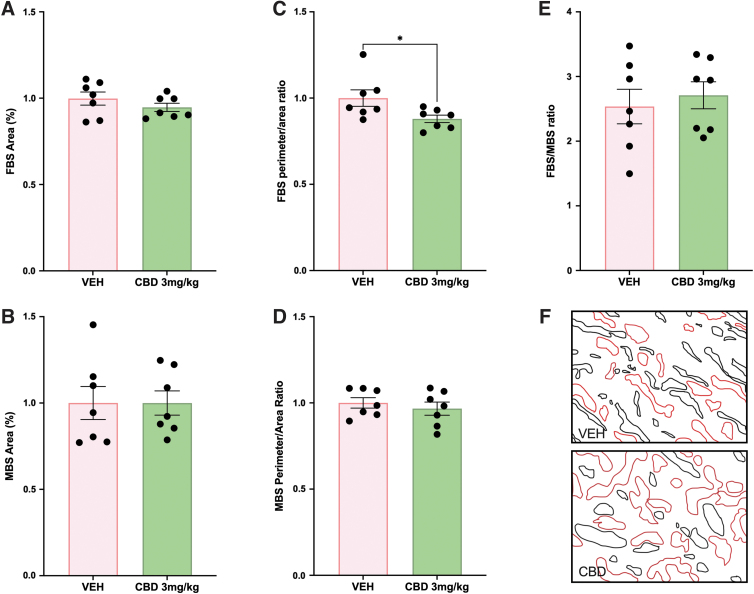
Exposure to 3 mg/kg CBD during gestation leads to a reduced perimeter:area ratio in the fetal capillaries of the labyrinth at E19.5 compared with the VEH control. **(A)** FBS area in the labyrinth layer. **(B)** MBS area in the labyrinth layer. **(C)** Perimeter:area ratio of the fetal blood spaces in the labyrinth layer. **(D)** The perimeter area ratio of the fetal blood spaces in the labyrinth layer. **(E)** Fetal blood space-to-maternal blood space ratio in the labyrinth layer. **(F)** Mask of fetal blood spaces (black) and maternal blood spaces (red) from representative histological image (400×magnification). Graphs present mean±SEM. Significance: Student's *t*-test (*p<0.05). CBD, cannabidiol; FBS, fetal blood space; MBS, maternal blood space; SEM, standard error of the mean; VEH, vehicle.

Although there were no changes in the relative size of the placental layers, the placental labyrinth as the site of maternal–fetal exchange was further assessed, given that changes in vascular development can be associated with limited fetal growth.^39^ Specifically, the fetal capillary network and the maternal blood sinusoids within the labyrinth were assessed to explore whether the FGR observed in the CBD-exposed pups may be attributed to placental insufficiency. The area of blood spaces, maternal–fetal blood space ratio and the perimeter-to-area ratio were measured as indicators of the surface available for nutrient exchange.^19,28–31^ Neither the fetal nor the maternal blood space area was altered in the placental labyrinth from CBD-exposed pregnancies compared with VEH control (Fig. 1A, B, F). However, the fetal blood space perimeter-to-area ratio was reduced in the CBD-exposed placentae (*p*=0.0405; Fig. 1C, F). Despite the perimeter-to-area change in the fetal blood spaces, there was no change to the perimeter-to-area ratio in the maternal blood spaces (Fig. 1D, F), nor was there a change to the fetal blood space-to-maternal blood space ratio (Fig. 1E, F).

### SynTII and vascular endothelial cell populations are reduced in the CBD-exposed labyrinth

**FIG. 2.**
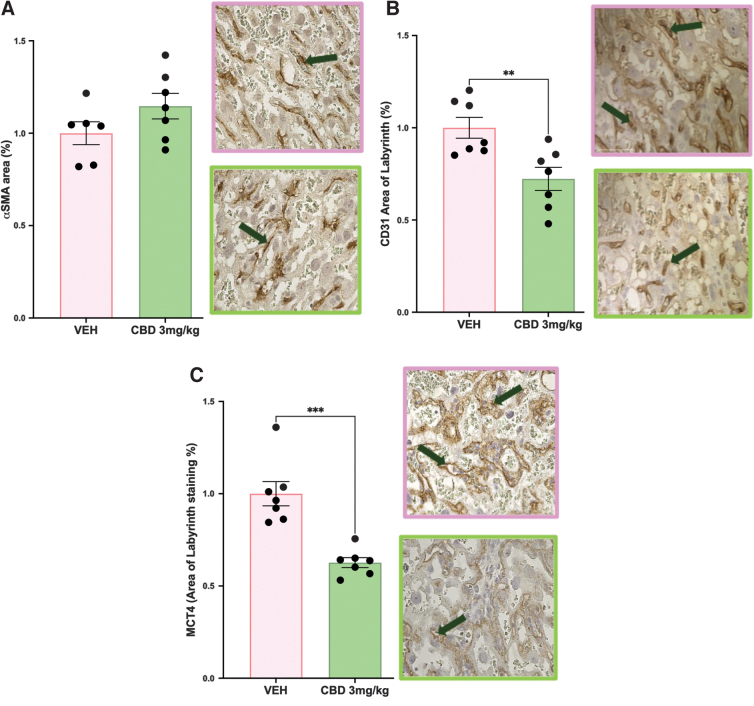
*In utero* exposure to 3 mg/kg CBD leads to reduced labyrinth endothelial and SynTII populations at E19.5 compared with VEH control. **(A)** αSMA+pericyte area in the labyrinth layer. **(B)** CD31+endothelial area in the labyrinth layer. **(C)** MCT4+SynTII area in the labyrinth layer. Histological representation images 400×magnification, green arrows identify positive staining. Graphs present mean±SEM. Significance: Student's *t*-test (**p<0.01, ***p<0.001). SynT-II, syncytiotrophoblast layer II.

Fetal blood spaces in the rodent labyrinth layer are lined with fetal endothelial cells and wrapped with pericyte cells that are in contact with SynTII cells.^40^ As such, with a change to the perimeter-to-area ratio in the fetal blood spaces, these three populations were assessed to see if they were altered. The increased αSMA pericyte staining was not significant in the placentae from CBD-exposed pregnancies (Fig. 2A). Assessment of both the CD31-positive endothelial cells and the MCT4-positive SynTII cells further revealed that both populations were significantly reduced in the CBD placentae compared with the VEH control placentae (*p*=0.0022 and *p*=0.0002, respectively, Fig. 2B, C).

We have previously demonstrated in other mouse models associated with altered αSMA pericyte expression that there can be a corresponding change to labyrinth extracellular matrix components.^19,28,41^ With no change in pericyte staining, labyrinth fibronectin and laminin assessment were as expected, unchanged between the placentae from the CBD and VEH-exposed pregnancies (data not shown).

### CBD-exposed placentae have altered expression of glucose transporters

Glucose transport is critical to a healthy pregnancy, with changes to the expression of placental glucose transporters reported in both human FGR and animal models of FGR.^19,42–46^ Fetal glucose uptake is dependent on successful transport across the placental interhemal membrane via the members of the glucose transporter family (GLUTs), which are regulated by the GR in the placenta.^47,48^ With glucose transporters localized to the site of maternal–fetal exchange in both the rodent and the human, it is logical that reduced GLUT1 expression is associated with FGR.^19,49,50^ Whereas in human pregnancy, GLUT1 is the primary glucose transporter, in rodent pregnancies, both Glut1 and Glut3 are responsible for placental glucose transport.^51^

**FIG. 3.**
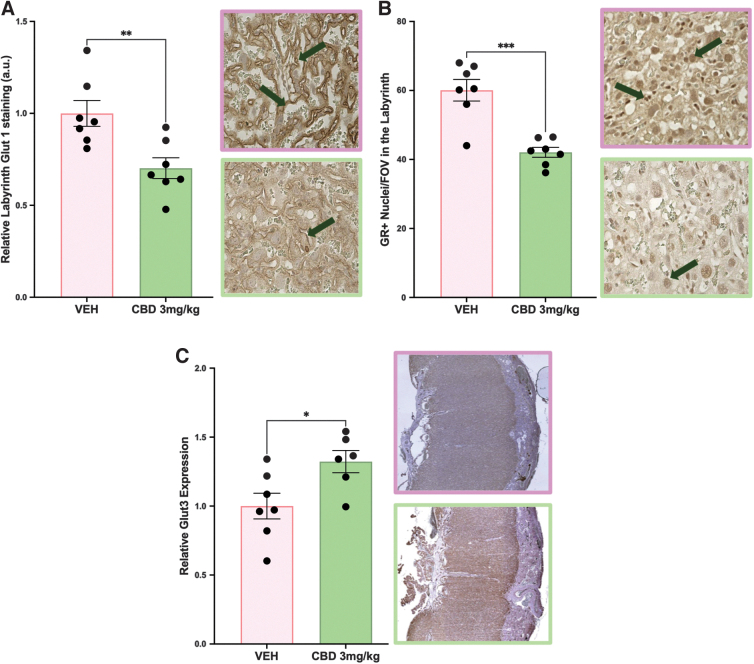
*In utero* exposure to 3 mg/kg CBD alters glucose transporters at E19.5 compared with VEH control. **(A)** Relative labyrinth Glut1 staining. **(B)** Labyrinth GR^+^ nuclei. **(C)** Relative placental Glut3 staining. Histological representation images 400×magnification **(A, B)**; 40×magnification **(C)**, green arrows identify positive staining. Graphs present mean±SEM. Significance: Student's *t*-test (*p<0.05, **p<0.01, ***p<0.001). GR, glucocorticoid receptor.

Therefore, we assessed the effects of gestational CBD exposure on placental Glut1, Glut3, and GR. Placentae from CBD-exposed pregnancies had reduced Glut1 and GR expression in the labyrinth (*p*=0.0062 and *p*=0.0002, respectively; Fig. 3A, B), with neither changed in the junctional zone (data not shown), compared with VEH control placentae. Conversely, Glut3 expression was increased in CBD placentae compared with VEH control (*p*=0.0259; Fig. 3C).

### Bulk RNAseq analysis revealed downregulated angiogenic pathways and upregulated metabolic pathways in the CBD-exposed placentae

**FIG. 4.**
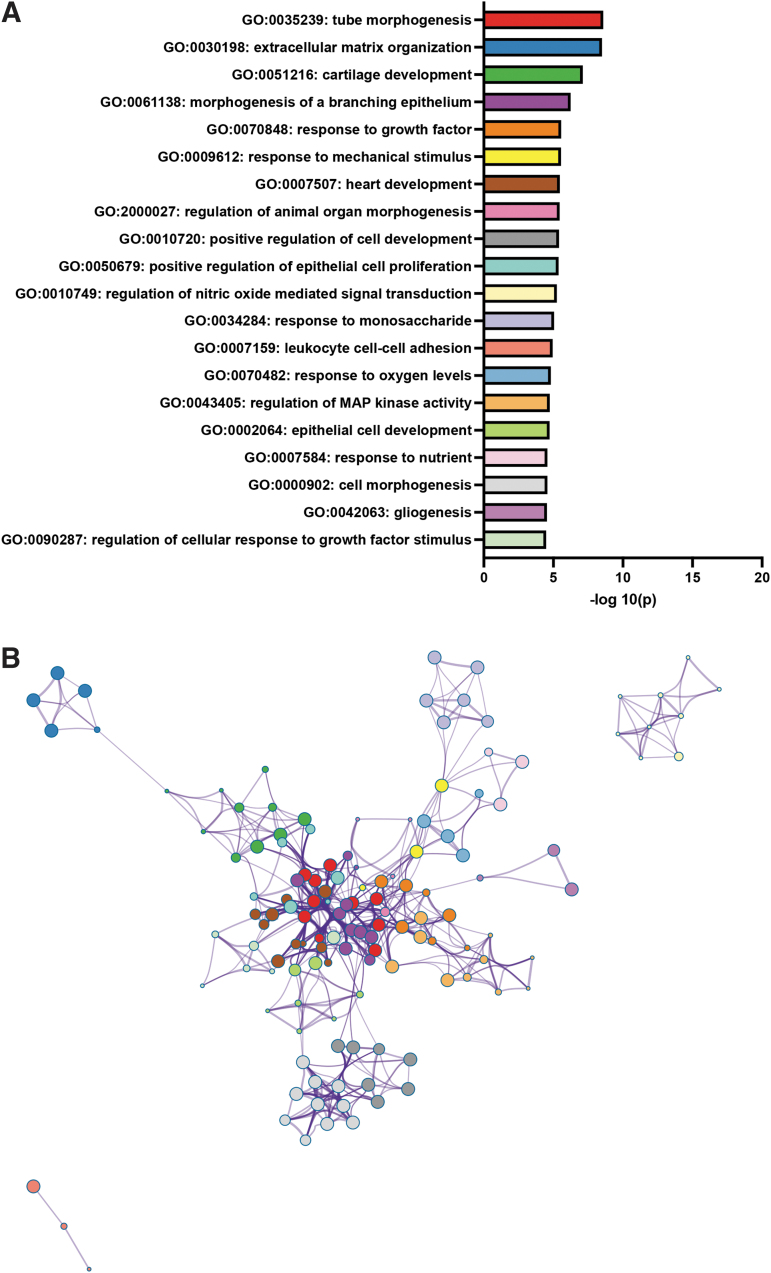
Downregulated GO biological processes in placenta from 3 mg/kg CBD exposed pregnancies compared with placentae from VEH control pregnancies. **(A)** −Log_10_(*p*-value) of downregulated GO biological process in the CBD versus VEH placenta. **(B)** Enriched ontology clusters: A subset of representative terms from each of the full GO clusters converted to a network layout. Each term is represented by a colored node **(**matching the graph in **A)**, with nodes of the same color belonging to the same GO cluster. Node size is proportional to the number of input genes that fall under the term. Terms with a similarity score >0.3 are linked by an edge (the thickness of the edge represents the similarity score). GO, gene ontology.

**FIG. 5.**
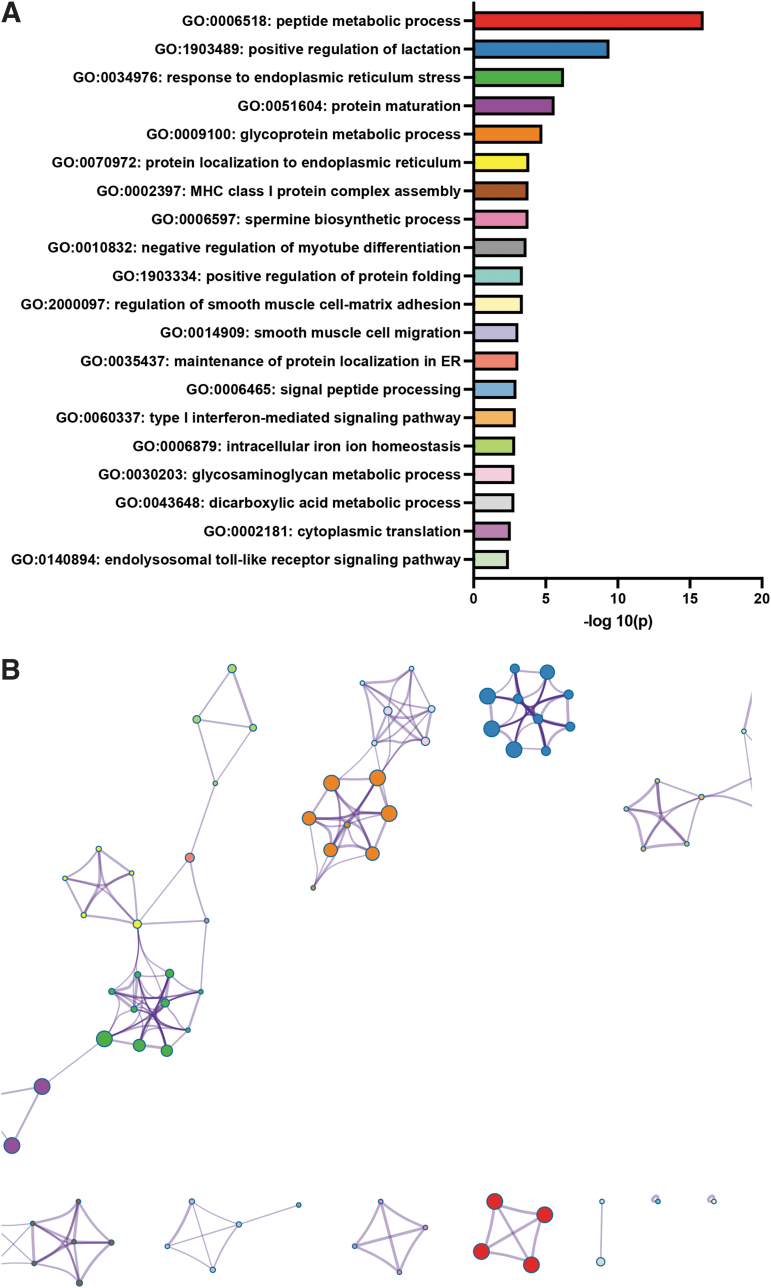
Upregulated GO biological processes in placenta from 3 mg/kg CBD-exposed pregnancies compared with placentae from VEH control pregnancies. **(A)** −Log_10_(*p*-value) of upregulated GO biological process in the CBD versus VEH placenta. **(B)** Enriched ontology clusters: A subset of representative terms from each of the full GO clusters converted to a network layout. Each term is represented by a colored node **(**matching the graph in **A)**, with nodes of the same color belonging to the same GO cluster. Node size is proportional to the number of input genes that fall under the term. Terms with a similarity score >0.3 are linked by an edge (the thickness of the edge represents the similarity score).

Bulk RNAseq analysis results were used to identify the most differentially expressed genes and the most up- and downregulated GO biological processes. Using the parameters of a 1.5-fold or greater change and an FDR corrected *p*-value of ≤0.05, 538 genes were identified as downregulated, and 865 genes were identified as upregulated. Using the list of downregulated genes, statistically enriched GO biological process terms were identified, and significant terms were hierarchically clustered (Fig. 4 and Supplementary Data S1 for the complete downregulated list of enriched terms). The same process was repeated with upregulated genes (Fig. 5 and Supplementary Data S2 for the complete upregulated list of enriched terms). Relevant to our histological analysis, the results revealed the downregulation of angiogenic and blood vessel formation biological processes and the mitogen activated protein (MAP) kinase activity biological process pathway cluster, with upregulation of four different metabolic pathways as well as an endoplasmic reticulum stress pathway (Figs. 4 and 5 and Supplementary Data S1 and S2).

Because altered placental development and functions can be attributed to changes in cell populations, once GO pathways were assessed, the bulk RNA seq data were used to look at the expression of genes typically associated with the placental labyrinth populations, including markers of trophoblast stem cells, labyrinth progenitors and labyrinth-specific cell populations, junctional zone progenitors and junctional zone-specific trophoblast populations. Using the unique profiles of the different population(s), we aimed to assess whether the bulk RNA seq data matched the histological findings. Trophoblast populations: Although trophoblast stem cell populations were not assessed histologically, the genes associated with these cell types indicated that most markers were either not significantly altered, or if they were, they were below the 1.5-fold threshold, except for *Esrrb* and *Sox2*, which were upregulated in the CBD-exposed placentae (Table 3). Among the markers of trophoblast progenitor populations, neither markers of labyrinth progenitors (*Epcam*) nor junctional zone progenitors (*Ascl2*) were differentially expressed. Within the labyrinth, markers of the SynTII population three of the genes frequently used to identify this population (*Gcm1, Synb*, and *Slc16a3/*MCT4)^52^ were significantly reduced; however, only *Gcm1* was reduced below the 1.5-fold threshold. The SynTI layer was not histologically assessed; although based on expression (*Epha4*, *Prkce, Slc16a1*/MCT1, *Snap91*, *Tgfa*),^52^ the bulk RNA seq results suggest that this layer was not altered as none of these genes were significantly changed. Moreover, three of four markers of S-TGC^52^ were not significantly altered (*Ctsq, Pparg*, and *Lepr*), whereas *Lifr* was significantly reduced but not above the 1.5-fold threshold. Within the junctional zone, the gene associated with P-TGCs (*Prl2c2*)^53^ was not differentially expressed. Furthermore, four of the five genes associated with spongiotrophoblast (*Prl5a1; Prl2b1; Prl2c1*; *Prl3b1*)^53^ were either not significantly altered or below the 1.5-fold threshold, with *Prl3a1*,^53^ significantly upregulated. Similarly, the expression of genes associated with the glycogen trophoblast (GlyT) populations^52^ had one marker significantly upregulated (*Prl6a1*), whereas the remaining four (*Aldh1a3, Pcdh12, Prl2a1* and *Prl7b1*)^53^ markers were either not differentially expressed or not above a 1.5-fold change. However, three genes that are expressed by both GlyT and SpT (*Tpbpa, Prl4a1*, and *Prl8a9*)^52,53^ were significantly upregulated, whereas *Prl7d1* was not significantly altered. Bulk RNA seq results indicated no significant change to four of five pericyte markers^54^ (*Acta2*/aSMA, *Cspg4*, *Des*, and *Rgs5*), although *Pdgfrb* was significantly upregulated. Expression of both markers of vascular endothelial cells (*Pecam1* and *Tek*) was significantly reduced and both were below the 1.5-fold threshold.

**Table 3.** Change in expression of genes associated with placental populations in the cannabidiol exposed placentae compared with the vehicle control placenta, based on bulk RNA seq results

**Gene name**	***p***	**Fold change**	**Cell type**
*Cdx2*	1.57E-01	1.23	TS cell
***Elf5***	**2.81E-02**	**−1.47**	**TS cell**
***Eomes***	**1.17E-02**	**−1.45**	TS cell
***Esrrb***	**3.00E-03**	*2.78*	**TS cell**
*Gata3*	2.26E-01	**−**1.08	TS cell
***Sox2***	**1.32E-02**	*2.39*	**TS cell**
*Tead4*	4.54E-01	**−**1.09	TS cell
***Tfap2c***	**1.14E-02**	**1.35**	**TS cell**
*Epcam*	3.86E-01	1.06	TB Lab progenitor
***Gcm1***	**2.27E-04**	***−1.64***	**SynTII**
***Synb***	**1.12E-02**	**−1.40**	**SynTII**
*Slc16a3*	0.00E-02	1.25	SynTII
*Epha4*	9.24E-02	**−**1.47	SynT1
*Eps8*	1.46E-01	1.14	SynT1
*Prkce*	2.64E-02	**−**1.30	SynT1
*Slc16a1*	4.33E-01	**−**1.13	SynT1
*Snap91*	1.21E-01	**−**1.34	SynT1
*Tgfa*	1.40E-01	1.77	SynT1
*Ctsq*	—	—	S-TGC
*Pparg*	1.50E-01	**−**1.14	S-TGC
*Lepr*	2.35E-01	**−**1.24	S-TGC
*Lifr*	3.02E-02	**−**1.48	S-TGC
*Ascl2*	1.83E-01	**−**1.42	JZ progenitor
*Prl2c2*	—	—	P-TGC
*Prl5a1*	2.28E-01	1.42	SpT
*Prl2b1*	9.93E-01	**−**1.00	SpT
***Prl3a1***	**5.53E-03**	*3.79*	**SpT**
*Prl2c1*	2.49E-01	**−**1.69	SpT
*Prl3b1*	6.31E-01	1.05	SpT
***Aldh1a3***	**4.55E-02**	**1.34**	**GlyT**
*Pcdh12*	8.79E-02	1.23	GlyT
*Prl2a1*	7.03E-01	**−**1.05	GlyT
***Prl6a1***	**2.27E-03**	*3.12*	**GlyT**
*Prl7b1*	1.83E-01	**−**1.76	GlyT
***Tpbpa***	**2.46E-03**	*3.15*	**SpT+GlyT**
***Prl4a1***	**3.21E-03**	*2.63*	**SpT+GlyT**
***Prl8a9***	**9.96E-03**	*2.50*	**SpT+GlyT**
*Prl7d1*	4.15E-01	1.37	SpT+GlyT
***Acta2***	**5.96E-02**	**−1.25**	**Pericytes**
*Cpsg4*	7.43E-01	**−**1.07	Pericytes
*Des*	8.94E-01	1.05	Pericytes
***Pdgfrb***	**4.07E-03**	*1.64*	**Pericytes**
*Rgs5*	1.07E-02	**−**1.27	Pericytes
***Pecam1***	**3.67E-02**	**−1.23**	**Vascular endothelial**
***Tek***	**1.49E-02**	**−1.41**	**Vascular endothelial**

Bold font identifies significance; italics identifies upregulated expression; bold italics identifies downregulated expression.

GlyT, glycogen trophoblast; S-TGC, sinusoidal trophoblast giant cells; SynT-II, syncytiotrophoblast layer II.

## Discussion

Epidemiological studies link *in utero* cannabis exposure to low-birth-weight outcomes; however, there are limited data on whether the individual cannabis components underlie FGR. In the rat, we previously demonstrated that postnatal day 1 pups from pregnancies exposed to Δ^9^-THC (3 mg/kg) have reduced fetal weight. However, whether the same dose and route of exposure to CBD impact fetal growth remained unknown. To our knowledge, this is the first study to demonstrate that at E19.5, fetuses from CBD-exposed pregnancies (3 mg/kg) are 10% smaller than those from the VEH control group. We previously showed that prenatal Δ^9^-THC (3 mg/kg) induced labyrinth-specific alterations in maternal and fetal blood space with decreased expression of the glucose transporter, Glut1. This study has similarly identified changes to fetal blood space perimeter-to-area ratio, altered glucose transporters, and additionally identified reduced fetal endothelial and SynTII populations in the CBD-exposed rat pregnancy. This is of significance as the fetal endothelial and SynTII populations are associated with the fetal blood spaces and express glucose transporters.^55^ Furthermore, transcriptomic analysis revealed significant upregulation of metabolic pathways in the CBD placentae. With no significant change in fetal demise, this dose and delivery method in the rat may prove useful in addressing the direct contributions of CBD on fetal development, including further placental and postnatal metabolic outcomes. This is relevant, considering recent clinical studies indicate that children of mothers who used cannabis in pregnancy exhibited dysglycemia and dyslipidemia as early as 5 years of age, even after controlling for socioeconomic status, ethnicity, tobacco use, and breastfeeding.^56^

The placenta from CBD-exposed pregnancies exhibited a decreased perimeter:area ratio in the fetal capillaries in the placental labyrinth and reduced CD31 staining (endothelial cells), which may suggest a defect in blood vessel formation and compromised angiogenesis.^57^ Angiogenesis is a tightly regulated process that is critical to placental development, with the role of the endothelial cell multifaceted in that they require successful chemotactic migration, invasion, proliferation, and differentiation into tubular capillaries, together with the production of a basement membrane around the vessels.^58^ The extracellular matrix components of the basement membrane around the vessels appeared unchanged in the CBD-exposed placentae; however, the bulk RNA seq analysis results support compromised angiogenesis and blood vessel formation. Specifically, downregulated biological processes included tube morphogenesis, angiogenesis, blood vessel morphogenesis, blood vessel development, vasculature development, chemotaxis, and locomotion. The role of CBD in this placental pathology is supported by studies demonstrating that CBD alters angiogenesis via multiple mechanisms.^58^

Specifically, using human umbilical vein endothelial cells (HUVECs) as an endothelial model, Solinas et al. demonstrated that CBD, in a concentration-dependent manner, inhibited HUVEC proliferation without inducing toxicity or apoptosis.^58^ Furthermore, they demonstrated that CBD inhibited HUVEC migration and proposed that reduced secretion of MMP2 may be an underlying contributor. Finally, using both an *in vitro* HUVEC spheroid model and an *in vivo* angiogenesis sponge model, their results indicate that CBD inhibits vascular endothelial growth factor (VEGF)-induced outgrowth of capillary-like structures, concluding that CBD inhibits sprouting of new capillaries in a dose-dependent manner.^58^ This suggests that in this study, *in utero* CBD exposure directly affected the endothelial population, thus indirectly affecting blood vessel formation and angiogenesis. Whether the reduced vascular endothelial populations and/or altered perimeter:area ratio of the fetal vessels in our model was owing to a change in migration or response to VEGF signaling remains to be explored.

The SynTII cell population was also reduced in the placentae from CBD-exposed pregnancies. The histological analysis identified reduced MCT4 staining, whereas bulk RNA seq analysis revealed reduced *Gcm1* and *Synb*. SynTII cells are closest to the fetal vasculature and express *Gcm1*. This is interesting considering that homozygous deletion of Gcm1 is embryonic lethal with failed SynT differentiation and compromised labyrinth development.^59,60^ In addition, placentae that only have one functional Gcm1 allele have SynTII abnormalities and evidence of SynT necrosis.^61^ Thus, it is possible that the reduced *Gcm1* may underlie the reduced MCT4 SynTII population. It is also important to note that cAMP, MAPK, and Wnt signaling pathways stimulate trophoblast cell fusion by activating the GCM1 transcription factor, which mediates the expression of SYNB, which is required for syncytialization. Supporting the involvement of this pathway is that our bulk RNA seq results identified the downregulation of the MAP kinase activity GO biological process pathway cluster. Collectively, the downregulation of both *Gcm1* and the MAP kinase activity pathway supports the idea that syncytialization of the SynTII cells was compromised in CBD-exposed placentae. However, whether this was a direct effect of CBD or an indirect signaling effect is unknown.

As SynTII cells are migratory, we used the transcriptomic results to explore additional mechanisms that may have compromised the size of the population. Some migratory cells require epithelial–mesenchymal transition (EMT) for this process; however, SynTII studies suggest that they do not undergo EMT; rather, their migration depends on a hepatocyte growth factor (HGF)/c-MET signaling axis.^62^ As SynT and vascular endothelial cells produce both HGF and c-MET,^63^ it is tempting to speculate that the reduced SynTII and vascular endothelial populations may lead to compromised (HGF)/c-MET signaling in CBD placentae. Bulk RNA seq results support this speculation as *Hgf* was downregulated in the CBD-exposed placentae (−1.93-fold-change; *p*=0.0148). Furthermore, HGF has been shown in mouse trophoblast stem cells to promote differentiation to SynT cells, whereas c-MET inhibits HGF-driven differentiation.^63^ Together these findings suggest that alterations in trophoblast differentiation and migration pathways may additionally contribute to the compromised SynTII layer in the CBD-exposed placentae.

CBD has recently been demonstrated to suppress angiogenesis via the downregulation of HIF1α expression. Specifically, CBD decreases HIF1α by upregulating its ubiquitination.^64^ HIF1α nor its ubiquitination were assessed in this study, although *Hif1α* was downregulated in the placentae from CBD-exposed placenta (−1.23-fold-change; *p*=0.0093). As such, CBD may, directly and indirectly, affect labyrinth vascular development and angiogenesis.

As the same populations contributing to the fetal vasculature are responsible for glucose transport, it is unsurprising that Glut1 and GR were both reduced in the CBD-exposed placenta. FGR in human pregnancies is associated with reduced placental GLUT1 expression^65^ and was speculated to contribute to the FGR identified in the rat Δ^9^-THC-exposed pregnancies. Unlike GLUT1, GLUT3 placental expression increases in human pregnancies associated with FGR.^65,66^ Consistent with those data, our CBD-exposed placentae had elevated Glut3 expression. Although it may seem counterintuitive to have elevated Glut3 associated with FGR, some possible mechanisms have been proposed: It is hypothesized that reduced GLUT1 protein expression triggers a compensatory mechanism to sustain fetal carbohydrate supply via increased GLUT3 expression. An alternate suggestion is that the GLUT3 is upregulated to cover the increased metabolic demands of the cells of the placenta.^65,67^

Supporting the idea that the metabolism in the CBD placentae is altered, the upregulated GO biological process clusters include the peptide metabolic process, glycoprotein metabolic process, glycosaminoglycan metabolic process, and tetrahydrofolate metabolic process. This suggests, similar to THC-exposed placentae, that the reduced placental Glut1 may contribute to the reduced fetal growth at E19.5 in CBD pregnancies, whereas increased Glut3 may indicate an altered metabolic state. Whether this adaptation would compensate for the fetus's needs by parturition requires further exploration.

Limitations of this study include i.p. injection as the route of delivery when injection is not the most common method of cannabis use. However, using i.p. delivery did allow for a direct comparison between our previous THC study and this study. As the delivery method can alter metabolism (reviewed in Rokeby et al.^24^), it will be important that future studies expand to include assessments of the delivery method of CBD. Similarly, this study only included one CBD dose over one long window of exposure. Furthermore, this study did not differentiate between placentae from male or female offspring. There is abundant evidence that male and female fetuses can respond differently to *in utero* stressors. As such, it is imperative that sex, a broader range of doses, different windows and lengths of exposure, paternal exposure and exposure during lactation are also evaluated. Finally, although our transcriptomics analysis allowed for the identification of up- and downregulated pathways that complimented our histological analysis, functional analysis was limited. Specifically, metabolic studies will be required to assess the effect of CBD on the metabolomics of the placentae and that of the individual cell populations.

## Conclusions

To the best of our knowledge, this is the first study to show that 3 mg/kg CBD exposure during rat pregnancy reduces fetal growth by ∼10% at E19.5. Like Δ^9^-THC, CBD altered the fetal capillary network in the placenta. The vascular endothelial and SynTII cells were most affected, and results suggest that these smaller populations may underlie the reduced expression of the Glut1 transporter and FGR. This study suggests that pregnant people should seek the advice of their physicians before using CBD during pregnancy. Furthermore, determining whether there are safer exposure windows, as CBD is being actively promoted as a treatment for many conditions that affect pregnant people, should be paramount.

## Abbreviations Used

Δ^9^-THC: Δ^9^-tetrahydrocannabinol
CBD: cannabidiol
E: embryonic day
EMT: epithelial–mesenchymal transition
FBS: fetal blood space
FDR: false discovery rate
FGR: fetal growth restriction
FOV: field of view
GlyT: glycogen trophoblast
GO: gene ontology
GR: glucocorticoid receptor
HGF: hepatocyte growth factor
HUVEC: human umbilical vein endothelial cell
i.p.: intraperitoneal
IHC: immunohistochemistry
MAP: mitogen activated protein
MBS: maternal blood space
SEM: standard error of the mean
S-TGC: sinusoidal trophoblast giant cells
SynT-II: syncytiotrophoblast layer II
TGC: trophoblast giant cells
VEGF: vascular endothelial growth factor
VEH: vehicle
